# Can Denosumab be used in combination with Doxorubicin in Osteosarcoma?

**DOI:** 10.18632/oncotarget.27669

**Published:** 2020-07-14

**Authors:** Francesca Punzo, Chiara Tortora, Maura Argenziano, Daniela Di Pinto, Elvira Pota, Martina Di Martino, Alessandra Di Paola, Francesca Rossi

**Affiliations:** ^1^Department of Woman, Child and General and Specialist Surgery, University of Campania “Luigi Vanvitelli”, Naples, Italy; ^2^Department of Experimental Medicine, University of Campania “Luigi Vanvitelli”, Naples, Italy

**Keywords:** osteosarcoma, Denosumab, Doxorubicin, RANK-L, bone mass loss

## Abstract

Osteosarcoma is an aggressive bone tumor of the pediatric age. It is therefore important to improve conventional therapies (chemotherapy and surgery). Anticancer drugs often cause osteoporosis due to a misbalance of RANK/RANK-L/OPG pathway.

Denosumab is a monoclonal antibody with high affinity and specificity to RANK-L, the ligand released by osteoblasts that enhances osteoclasts differentiation and bone resorption. It is used in osteoporosis and in other conditions characterized by bone mass loss. Doxorubicin is a chemotherapic drug used in several kinds of tumors, and also patients treated with it often develop osteoporosis.

We investigated the effects of Denosumab alone and in combination with Doxorubicin, in two human osteosarcoma cell lines (MG63 and U-2 OS). We evaluated the effect of these treatments on apoptosis, cell cycle progression, invasion capacity and bone metabolism.

We observed for the first time an anti-invasive effect of Denosumab in OS cells and confirmed its anti-osteoporotic activity also in Osteosarcoma. On the other hand, we demonstrate that Denosumab not only does not affect apoptosis and cell cycle progression, but when used in combination with Doxorubicin, it causes an unexpected reduction of its activity. These results indicate that the presence of Denosumab might inhibit the efficacy of the chemotherapic drug.

In conclusion, while our results certainly support and confirm the efficacy of Denosumab in Osteoporosis, we discourage the use of Denosumab in addition to conventional chemotherapy in Osteosarcoma, even though, certainly further investigations are necessary to better clarify the clinical role of this monoclonal antibody in cancer.

## INTRODUCTION

Osteosarcoma (OS), among bone tumors, is the most common in children and adolescents [1]. It has an high invasion and metastasis potential and causes alteration of bone metabolism and Osteoporosis (OP) with high risk of bone fractures in long-term survivors [2]. One of the most used anticancer drug to treat OS is Doxorubicin (Doxo) [3, 4]. It belongs to the anthracycline family of antibiotics. It acts inhibiting the synthesis of DNA and its repair in rapidly growing cells [5] and causing oxidative damage to cellular membranes, proteins and DNA, triggering apoptosis [6]. There is evidence that Doxo might negatively affect the skeleton and Doxo-treated children frequently reach a lower adult stature and have a higher fracture risk [7, 8]. Some *in vivo* studies report an increase in markers of bone resorption [7] and a decrease in bone turnover [9, 10]. *In vitro* studies show that Doxo inhibits osteoblasts (OBs) proliferation, differentiation and mineralization while stimulates osteoclasts (OCs) differentiation [11, 12]. The bone remodeling process is maintained by a fine balance between bone resorption by OCs and new bone deposition by OBs. The receptor activator of nuclear factor κB ligand (RANK-L) is a protein highly expressed by OBs and able to induce OCs differentiation and activation by binding the Receptor Activator of Nuclear Factor κ-B (RANK), its specific receptor on OCs surface. This interaction causes bone resorption, which is counterbalanced by Osteoprotegerin (OPG), an OBs glycoprotein that works as antagonist of RANK by binding RANK-L and preventing the OCs activation [13]. In literature there are several evidences about the alterations in RANK-L and RANK expression levels in both OS cell lines and OS patients [14]. *In vitro* studies have shown a reduction in OS cells invasion by inhibiting RANK signaling pathway with OPG [15, 16]. Moreover, the overexpression of RANK-L in OS patients seems to be associated to poor response to chemotherapy and therefore to reduced survival [17, 18].

Denosumab (Den) is a human monoclonal antibody with high binding affinity and specificity to RANK-L. It exerts its action by limiting the OCs differentiation and activation, suppressing bone resorption, hence, contributing in increasing bone mineral density (BMD) [19–22]. For these reasons, Den is indicated for the treatment of several skeletal disorders characterized by bone mass loss [22, 23]. Above all, Den is crucial to treat OP. In OP patients it significantly increases BMD more than in individuals treated with bisphosphonates (BP) [24]. Other applications of Den are in patients with bone metastasis from solid tumors, in order to reduce the occurrence of skeletal fractures, as well as in patients with hypercalcemia due to Den capacity to reduce the calcium release by OCs [18, 25, 26]. Moreover Den finds application in the giant cell tumors, in osteogenesis imperfecta, in juvenile Paget disease and other benign fibro-osseous lesions [27–30].

In the last years we are actively investigating the effects of different compounds in order to identify new molecular targets to improve the efficacy of OS treatment and reduce its adverse effects [31–34]. Based on this need and seen the role of Den in bone mass loss conditions, we decided to investigate the effects of this human monoclonal antibody in bone metabolism of human derived OS cells expressing RANK/RANK-L (MG63 and U-2 OS) alone and in combination with Doxo. We evaluated apoptosis, cell cycle progression and OS cell migration, to consider if Den can be a valid aid in improving the outcome of OS patients and reduce OP incidence after chemotherapy.

## RESULTS

### RANK, RANK-L and OPG expression

**Figure 1:**
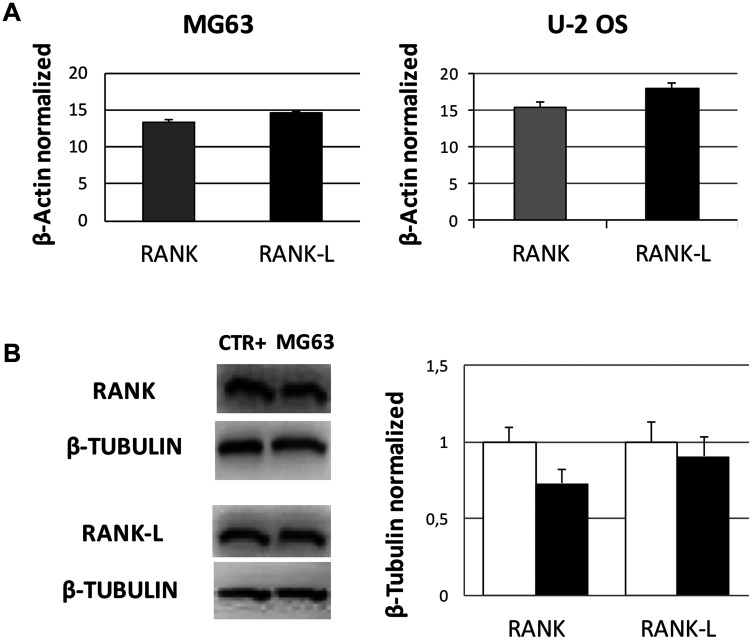
RANK and RANK-L mRNA and protein expression levels in OS cell lines. (**A**) RANK and RANK-L mRNA expression levels in MG63 cell line and in U-2 OS cell line determined by q-PCR and normalized for the housekeeping gene β-Actin. For each cell line, data are expressed as mean ± SD of three independent experiments. (**B**) RANK and RANKL protein expression levels in OS cells compared to positive control samples (CTR+), represented by human osteoclasts from healthy donors for the evaluation of RANK expression and by human osteoblasts from healthy donors for the evaluation of RANK-L expression, determined by WB, starting from 15 μg of total lysates. The graph represents both cell lines results, while the WB image displayed is the most representative one. The proteins were detected using Image Studio Digits software and the intensity ratios of immunoblots compared to CTR taken as 1 (arbitrary unit), were quantified after normalizing with respective loading controls for the housekeeping protein β-Tubulin. Histogram shows the relative quantification for RANK and RANK-L expression as mean ± SD of three independent experiments on both cell lines.

**Figure 2:**
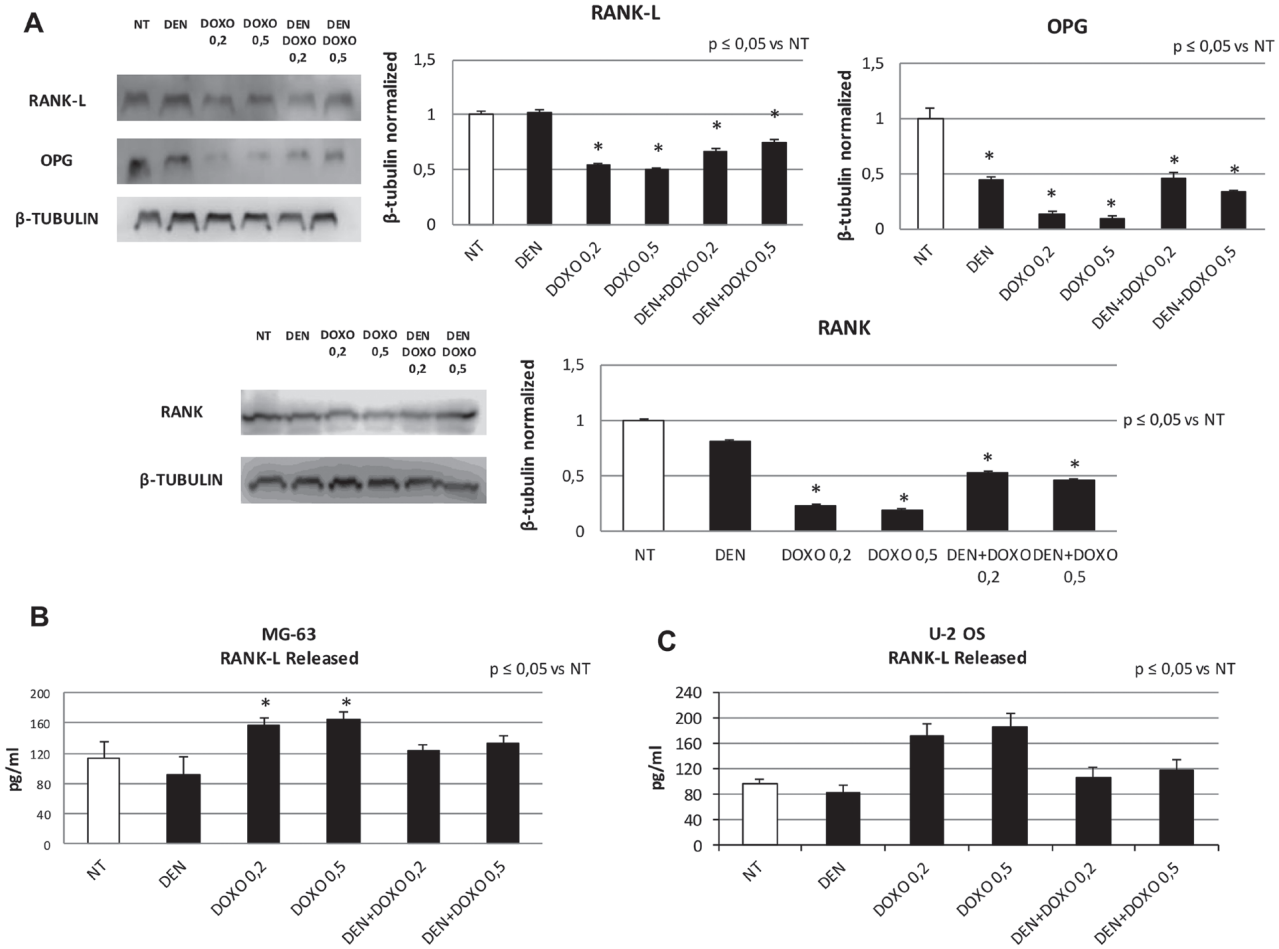
RANK, RANK-L and OPG protein expression levels and RANK-L released concentration after treatment with Den and Doxo in OS cell lines. (**A**) RANK-L, OPG and RANK protein expression levels in MG63 and U-2 OS cells determined by WB, starting from 15 μg of total lysates, after 48 h of exposure to Den [30 μg/mL] and Doxo [0,2/0,5 μM] alone or in combination. The most representative WB images are displayed. The proteins were detected using Image Studio Digits software and the intensity ratios of immunoblots compared to MG63 untreated (NT), taken as 1 (arbitrary unit), were quantified after normalizing with respective loading controls for the housekeeping protein β-Tubulin. Histograms show the relative quantification for RANK-L, OPG, RANK expression as mean ± SD of three independent experiments on both cell lines. A One-way analysis of variance (ANOVA), followed by Tukey’s post hoc test, has been used to evaluate differences in RANK-L and OPG expression among groups. ^*^indicates *p* ≤ 0.05 compared to the untreated control (NT). (**B**) The release of RANK-L from MG63 cells and from U-2 OS (**C**) cells determined by ELISA assay, after 48 h of exposure to Den [30 μg/mL] and Doxo [0,2/0,5 μM] alone or in combination. RANK-L concentration was determined on a standard concentration curve according to the manufacturer’s instructions. For each cell line the assay was conducted three times. Data are expressed as mean ± SD (pg/ml). A One-way analysis of variance (ANOVA), followed by Tukey’s post hoc test, has been used to evaluate differences in RANK-L release among groups. ^*^indicates *p* ≤ 0.05 compared to the untreated control (NT).

Prior to Den and Doxo treatments we evaluated by RT-qPCR and Western Blotting whether RANK and RANK-L were expressed in untreated OS cells. They are expressed as shown in Figure 1, in both cell lines. Hence, we treated MG63 and U-2 OS cells with Den [30 μg/mL] and Doxo [0,2 and 0,5 μM] and measured the effects on RANK and RANK-L at 48 h, by RT-qPCR and Western Blotting. Den does not induce any alteration in RANK and RANK-L expression levels. When treating OS cells with Doxo the expression of RANK-L was reduced and RANK and OPG were almost abolished when measuring them in cell lysate extracts (Figure 2A). The result was opposite when we measured RANK-L released (by the cells) in cell’s surnatant by ELISA Assay: Den treatment reduced RANK-L while Doxo increased it. The combined treatment with Den and Doxo reduced the difference in RANK-L release equal to the control level (Figure 2B and 2C).

### Effects of treatments on OS cells migration capacity

**Figure 3:**
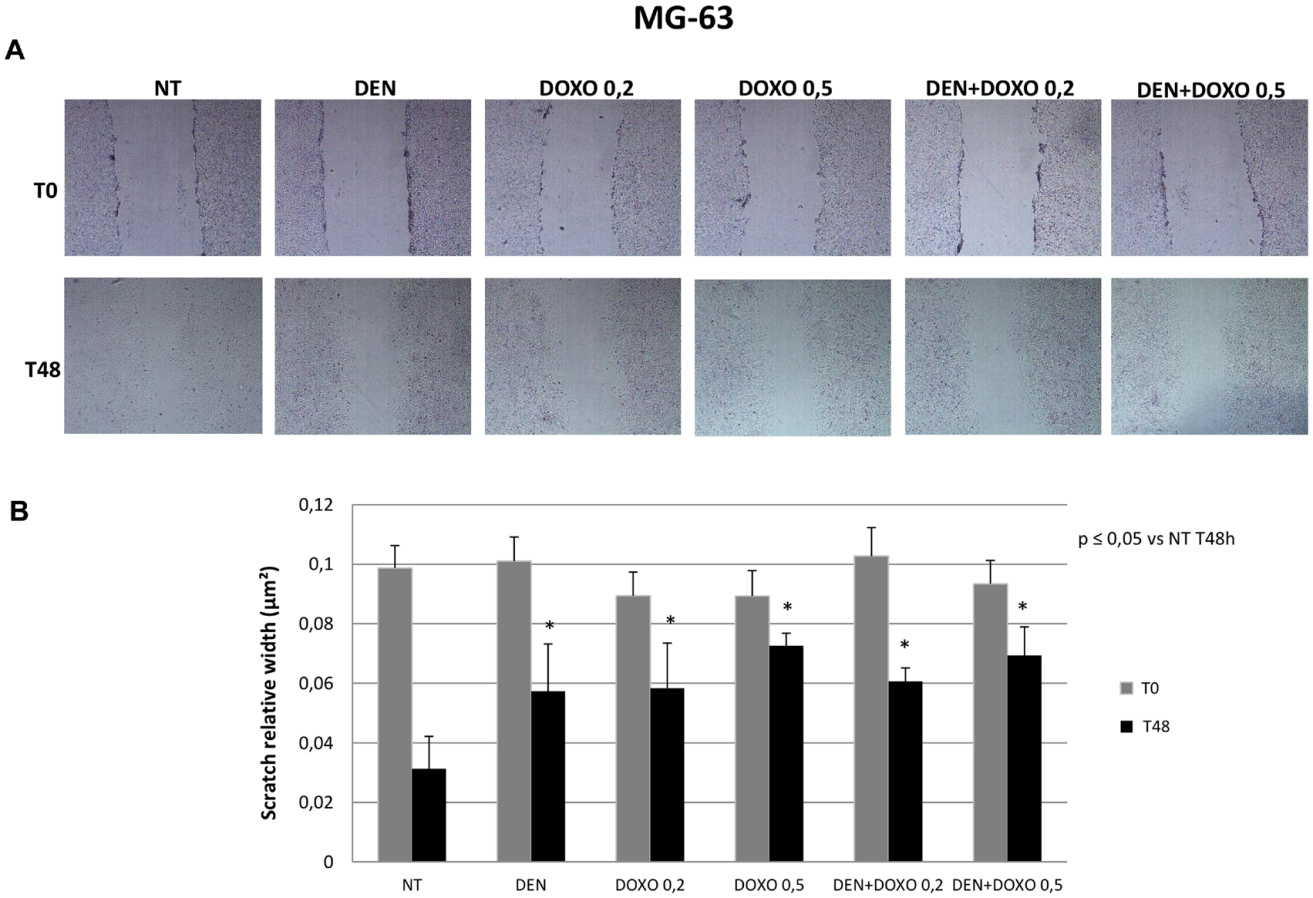
Invasive capacity evaluation in MG63 cells after treatment with Den and Doxo (Scratch Assay). (**A**) MG63 cells have been plated in a six well plate and a single scratch has been performed in the center of the wells at the confluence of 70–80%. Cells have been treated with Den [30 μg/mL] and Doxo [0,2/0,5 μM] alone or in combination for 48 h. The representative images, taken at T0 and T48 h, of scratch relative width (nm^2^) are displayed. Images were taken on a AE2000 inverted microscope at 4× magnification. (**B**) The Histogram displays the relative quantification of the scratch width (nm^2^) after 24 h treatment with Den [30 μg/mL] and Doxo [0,2/0,5 μM] alone or in combination, compared with the initial width area measured with Motic images plus 2.0 Software. Data derived from three different assays. A One-way analysis of variance (ANOVA), followed by Tukey’s post hoc test, has been used to evaluate the statistical differences among groups. ^*^indicates *p* ≤ 0.05 compared to the untreated control (NT).

**Figure 4:**
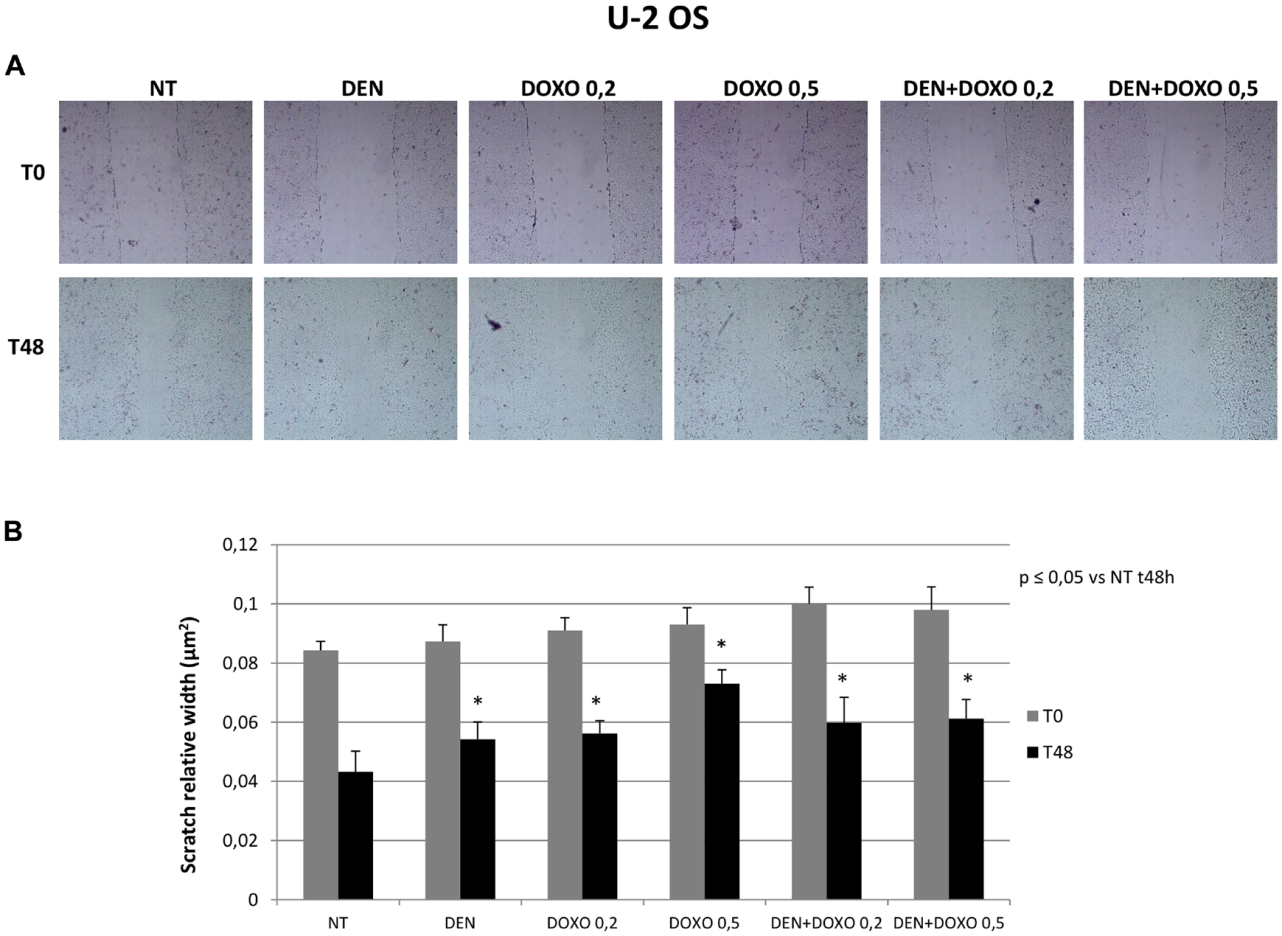
Invasive capacity evaluation in U-2 OS cells after treatment with Den and Doxo (scratch assay). (**A**) U-2 OS cells have been plated in a six well plate and a single scratch has been performed in the center of the wells at the confluence of 70–80%. Cells have been treated with Den [30 μg/mL] and Doxo [0,2/0,5 μM] alone or in combination for 48 h. The representative images, taken at T0 and T48 h, of scratch relative width (nm^2^) are displayed. Images were taken on a AE2000 inverted microscope at 4× magnification. (**B**) The Histogram displays the relative quantification of the scratch width (nm^2^) after 24 h treatment with Den [30 μg/mL] and Doxo [0,2/0,5 μM] alone or in combination, compared with the initial width area measured with Motic images plus 2.0 Software. Data derived from three different assays. A One-way analysis of variance (ANOVA), followed by Tukey’s post hoc test, has been used to evaluate the statistical differences among groups. ^*^indicates *p* ≤ 0.05 compared to the untreated control (NT).

Migration capacity has been measured in terms of reduction of the scratched area (nm^2^) 48 h after the scratch compared to the same well at time 0. The wider is the area 48 h later, the lower is the migration observed in the well. Scratch test demonstrated that OS cells treated with Den [30 μg/mL] as well as cells treated with Doxo [0,2 and 0,5 μM] and with the two compounds together, show a significant reduction of migration capacity compared to untreated cells and that this effect is demonstrated in both OS cell lines (MG63 in Figure 3 and U-2 OS in Figure 4).

### Effects of treatments on apoptosis in OS cells

**Figure 5:**
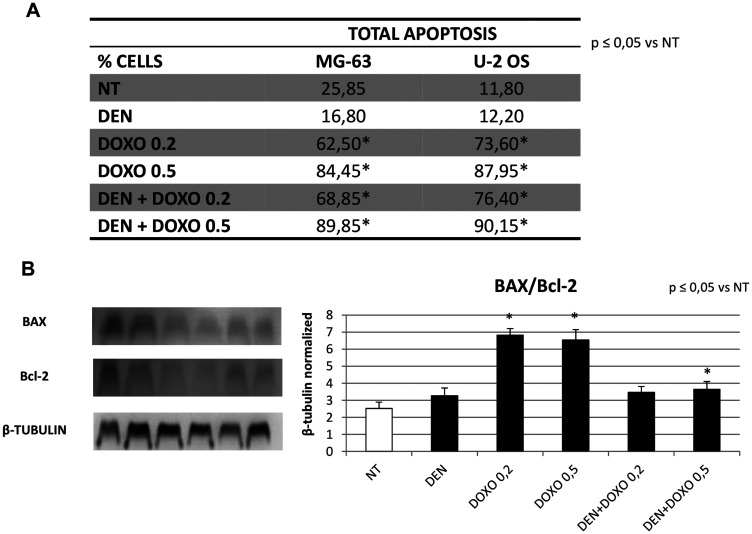
Apoptosis evaluation in OS cell lines after treatment with Den and Doxo determined by cytofluorimetric assay and by Western blot (BAX/Bcl-2 ratio). (**A**) Percentage of total apoptotic MG63 and U-2 OS cells treated with Den [30 μg/mL] and Doxo [0,2/0,5 μM] alone or in combination for 48 h. For each cell line, the results are presented as the mean percentage of three independent experiments and were analyzed by one-way analysis of variance (ANOVA) followed by Tukey’s post hoc test. ^*^indicates *p* ≤ 0.05 compared to the untreated control (NT). (**B**) BAX and Bcl-2 protein expression levels in OS cells, determined by WB, starting from 15 μg of total lysates after 48 h of exposure to Den [30 μg/mL] and Doxo [0,2/0,5 μM] alone or in combination. The most representative images are displayed. The proteins were detected using Image Studio Digits software and the intensity ratios of immunoblots compared to the untreated control, taken as 1 (arbitrary unit), were quantified after normalizing with respective loading controls for the housekeeping protein β-Tubulin. Histogram shows the ratio between BAX and Bcl-2 as the mean ± S.D. from three experiments. A One-way analysis of variance (ANOVA), followed by Tukey’s post hoc test, has been used to evaluate the statistical differences among groups. ^*^indicates *p* ≤ 0.05 compared to the untreated control (NT).

We studied the effect on apoptosis in MG63 and U-2 OS cells after treatment with Den [30 μg/mL] and Doxo [0,2 and 0,5 μM] alone and in combination by means of a citofluorometric assay on Muse Cell Analyzer (Merk-Millipore) and by Western Blot evaluating the BAX/Bcl-2 Ratio. Our results highlighted a statistically relevant increase in total apoptosis at 48 h with Doxo at increasing concentration as expected, and no increase with Den alone. The apoptosis observed at the citofluorimetric assay with Den and Doxo together resemble the same values observed with Doxo alone (Figure 5A). The BAX/Bcl-2 ratio showed an over 3-fold increase with Doxo alone but when combining the two compounds, the increase was reduced (Figure 5B).

### Effects of treatments on cell cycle progression in OS cells

**Figure 6:**
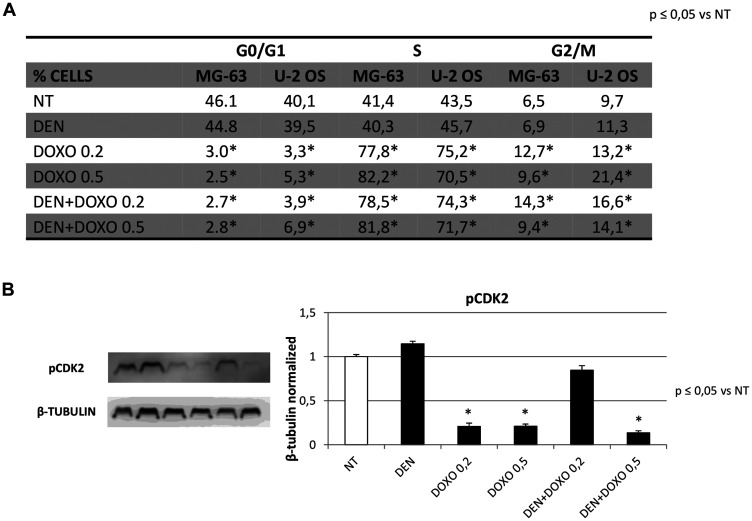
Cell Cycle progression in OS Cell lines before and after treatment with Den and Doxo by cytofluorimetric assay and Western Blotting (pCDK2). (**A**) Percentage of MG63 cells and U-2 OS cells at different phases of the cell cycle (G0/G1 phase, S phase and G2/M phase) after treatments with Den [30 ug/mL] and Doxo [0,2/0,5 μM] alone or in combination, for 48 h. Cells were harvested, fixed, incubated with Muse Cell Cycle reagents and analyzed on “MUSE Cell Analyzer”. (**B**) pCDK2 protein expression levels in OS cells were determined by Western Blot, starting from 15 mg of total lysates after 48 h of exposure to Den [30 ug/mL] and Doxo [0,2/0,5 μM] alone or in combination. The most representative images are displayed. The proteins were detected using Image Studio Digit software and the intensity ratios of immunoblots, compared to that of untreated control (NT), taken as 1 (arbitrary unit), were quantified after normalizing with respective loading controls for the housekeeping protein β-Tubulin. Histogram shows the relative quantification for pCDK2 expression as the mean ± S. D. from three experiments. A One-way analysis of variance (ANOVA), followed by Tukey’s post hoc test, has been used to evaluate the statistical differences among groups. ^*^indicates *p* ≤ 0.05 compared to the untreated control (NT).

To evaluate the possible role of Den alone and with Doxo in our two OS cell lines in cell cycle progression, we performed a specific assay on the Muse Cell Analyzer. Cells were exposed for 48 h to Den [30 μg/mL] and Doxo [0,2 and 0,5 μM]. The Muse cell Analyzer automatically displayed the percentage of cells in G0/G1-S and G2/M phases of the cell cycle which resulted not significantly different between Den treated cells and non-treated while, as expected, MG63 treated with Doxo or Doxo with Den, accumulated in S phase (Figure 6A). To confirm the effect of Den and Doxo on cell cycle progression, we also evaluated the expression levels of phosphorylated CDK2 protein (pCDK2). Den did not reduce the expression of this important kinase for cell cycle progression, 48 h after administration, while Doxo drastically reduced the protein, confirming its capacity to impair cell cycle progression. Moreover, we observed that when Doxo at [0,2 μM] is used in combination with Den, the reduction of pCDK2 seems impaired (Figure 6B).

## DISCUSSION

OS is an aggressive bone tumor of the pediatric age, characterized by an alteration of bone metabolism. The actual therapy for OS is surgery and chemotherapy [1]. Doxo is one of the most common first-line chemotherapeutic drug in numerous types of cancer, including OS [4]. Unfortunately, it leads to many side effects, such as OP with the risk of severe bone fractures [5, 7]. Hence the necessity to improve cancer therapy and counteract its negative effects on bone.

Bone tissue homeostasis is maintained by RANK/RANK-L/OPG pathway. RANK-L is a protein expressed by OBs that binds to its specific receptor, RANK, present on OCs precursors’ surface, inducing OCs differentiation and activation and, therefore, bone resorption. This effect is counterbalanced by OPG which binds to RANK-L inhibiting its interaction with RANK [13].

Recently, it has been demonstrated that RANK/RANK-L signaling is not only involved in bone remodeling, but it also plays a crucial role in tumorigenesis and metastasis of several kind of malignancies, such as breast tumor [35–37], therefore RANK-L inhibition could arrest the cells migration. In literature, several studies evaluated the possible anticancer effects mediated by RANK-L inhibitors [38]. In OS patients it has been shown that RANK and RANK-L are usually overexpressed and related to poor outcome [17].

Den is a human monoclonal antibody directed against the RANK-L. It mimics the action of OPG and limits OCs differentiation and activation, preventing bone resorption. Den is an approved and effective therapy for OP [22, 23, 39] and other skeletal disorders characterized by bone mass loss [27]. Since 2010 Den has been studied also as possible therapy to contrast cancer progression [19, 26]. Therefore, to improve the outcome and reduce the OP chemotherapy-related in OS patients expressing RANK, we treated two human OS derived cell lines (MG63 and U-2 OS) with Den. Our aim was to investigate its possible anti-cancer properties (by evaluating cell cycle progression, apoptosis, migration capacity) and to consider the possibility to extent the use of this antibody, in combination with traditional chemotherapy agents (Doxo).

Consistently with data present in literature, after Den treatment, we did not observe any variation in RANK and RANK-L levels, which allows us to assume that the equilibrium between RANK and RANK-L was not altered by this drug [38]. However, Den has an anti-invasive effect at the Scratch Test that seems not to be related to a direct action on RANK/RANK-L signaling, suggesting that other pathways are involved in its biological function and worth to be investigated. Doxo, strongly reduced RANK-L in OS cells and a consequently reduced their migration capacity as demonstrated in fact, also with the scratch test.

On the other hand, Doxo increased RANK-L release by OS cells. The RANK-L released, could stimulate the OCs present in the tumor microenvironment, increasing the bone resorption, favoring OP. Moreover, Doxo induces a strong reduction of OPG which is a physiologically important regulator in OCs differentiation and function. These data are in agreement with the well-known pro-osteoporotic effect of the chemotherapic treatments. Den in co-treatment with Doxo, seems to counteract the bone damage strongly increasing the OPG levels, however without restoring them, and reducing the release of RANK-L.

Den does not affect apoptosis, while Doxo confirmed its pro-apoptotic capacity even though, when used in combination with Den, we observed an unexpected reduction of its activity, indicating that the presence of Den might even inhibit the apoptotic activity of the chemotherapic drug. Moreover, we demonstrated that Doxo is able to block OS cells in S phase, and reduce the phosphorylated CDK2 protein but again, when Doxo is used at a lower dosage, in combination with Den, the reduction of pCDK2 seems impaired. Our study has been performed only on human cell lines, thus, it lacks of *in vivo* experiments that could confirm what observed *in vitro*. This limitation should be considered, although it does not impair the delicate clinical implications of the data presented.

In conclusion our study confirmed the well-known anti-osteoporotic activity of Den and, for the first time we observed an anti-invasive effect in MG63 and U-2 OS cell lines. Despite this important result, our initial hypothesis on the possible combined use of Den and Doxo has not been validated, which alerts us regards the clinical use of this drug in OS. In addition, in literature are described two case reports of a Giant Cell Tumor of bone treated with Den that developed in high grade OS [40, 41]. Therefore, while our results certainly support and confirm the efficacy of Den in OP, definitely further studies are necessary to clarify its clinical role in bone cancer.

## MATERIALS AND METHODS

### OS cell lines

MG63 and U-2 OS cell lines were purchased from Sigma Aldrich and cultured in EMEM medium (for MG63) and Mc Coy medium (for U-2 OS) with 1% Non-Essential Amino Acids (NEAA), 10% fetal bovine serum (FBS), supplemented with 100 U/ml penicillin (Gibco), 100 U/ml streptomycin (Gibco) and 2 mM L-glutamine (Euroclone). Cells were cultured at 37°C in a humidified atmosphere with 5% CO2. After 48-hours adhesion cells were harvested using trypsin, washed and counted on a microscope using a Burker Haemocytometer and 1,0 × 10^6^ per well were plated in a 6 multiwell. Once 80% confluence was reached, Den [30 μg/mL] and Doxo, at two different concentrations, [0,2 μM] and [0,5 μM], were added alone and in combination. Cells were harvested at 48 h for mRNA isolation, protein extraction, Muse^®^ “Annexin V and Dead Cell Assay” (Millipore), Muse^®^ “Cell Cycle Assay” (Millipore) and Scratch test.

### Drugs and treatments

Doxo is an anthracycline antibiotic, used as chemotherapeutic drug for the treatment of both solid and hematological malignancies. We used Doxorubicin hydrochloride commercialized as Adriblastin^®^ by Pfizer Italia S. r. l. We dissolved 50 mg of Doxo powder in 25 mL of sterile water, obtaining the final concentration of 2 mg/mL. Doxo was used on MG63 and U-2 OS cell lines at two different concentrations: [0,2 μM] and [0,5 μM].

Den is a human monoclonal IgG2 antibody specific for RANK-L and produced in Chinese hamster ovary cell line (CHO) by recombinant DNA technology. It inhibits the differentiation and the activation of osteoclasts principally indicated for post-menopausal OP. We used Den commercialized as XGEVA^®^ purchased from Amgen Inc. as a single-use vial containing 120 mg of Den powder. We dissolved the powder in the vial with 1,7 mL of sterile water obtaining a colorless solution with the final concentration [70 mg/mL]. Den was used on MG63 and U-2 OS cells at [30 μg/mL]. Non-treated cultured cells were maintained in incubation media during the relative treatment time with and without vehicle (sterile water).

### Total RNA extraction and reverse transcription quantitative polymerase chain reaction (RTqPCR)

Following treatment with Den [30 μg/mL] and Doxo at two different concentrations, [0,2 μM] and [0,5 μM], at 48 h OS cells were harvested. Cells without treatment served as the control group. The total RNA was extracted using Qiazol^®^ (Qiagen) according with the manufacturer’s instructions. SensiFast cDNA Synthesis Kit (Bioline) was used to synthesize, from 1000 ng mRNA, the first strand cDNA. The transcript levels of RANK and RANK-L genes were detected by RT-qPCR on a CFX96 Real-Time PCR system (Bio-Rad), loading 5 μl of cDNA diluted 1:10, 1,6 μl of Primers Mix [10 μM] each, 10 μl of I-Taq Universal SYBR^®^ Green Master Mix (Bio-Rad) and 3,4 μl H_2_O. The cycling conditions were 10 min. at 95°C (initial denaturation), followed by 40 cycles of 15 sec. at 94°C (denaturation) and 1 min at 68°C (annealing/extension/data collection). The β-Actin gene served as the reference gene for the normalization of the real-time PCR products. The PCR primers used to detect each gene were designed using Primer 3 program and synthesized by Sigma Aldrich (RANK-L_F 5′-TGGTGGATGGCTCATGGTTA-3′, RANK-L_R 5′-ATGGGATGTCGGTGGCATTA-3′, RANK_F 5′-GCAGCTCAACAAGGACACAG-3′ RANK_R 5′-AAGGTACAGTTGGTCCAGGG-3′, β-Actin_F 5′-GCGAGAAGATGACCCAGATC-3′, β-Actin_R 5′-GGATAGCACAGCCTGGATAG-3′). The linearity and efficiency of the assays were tested over dilutions of input cDNA spanning five orders of magnitude. Assays were performed in triplicate. The dissociation curve analysis of amplification products was performed at the end of each PCR reaction to confirm the specificity of the amplification. The 2^-ΔΔCt^ method was used to analyze the data and obtain the relative gene expression levels compared to the controls.

### Western blotting

Proteins were extracted from treated and non-treated cells using RIPA Lysis Buffer (Millipore) and following the manufacturer’s instructions. RANK, RANK-L, OPG, pCDK2, BAX, Bcl-2 proteins were characterized in total lysates from cell line cultures by Western blotting. Membranes were incubated overnight at 4°C with the following antibodies: anti-RANK L (1:100, BIOSS), anti-RANK (1:250, BIOSS), anti-OPG (1:500, Elabscience), anti-pCDK2 (1:1000, abcam), anti-BAX (1:200, Santa Cruz), anti-Bcl2 (1:100, Santa Cruz). Reactive bands were detected by chemiluminescence (Immobilon western Millipore) on a C-DiGit^®^ Blot Scanner (LI-COR Biosciences). A mouse monoclonal anti-β-Tubulin antibody (1:5000, Elabscience) was used to check for comparable protein loading and as a housekeeping protein. Images were captured, stored and analyzed using “Image studio Digits ver. 5.0” software.

### Cell dead and Annexin V assay

Apoptosis has been evaluated by a fluorimetric assay on the Muse cell analyzer machine with the “Cell dead and Annexin V Assay Kit”. Test was performed after 48 h of exposure to Den [30 μg/mL] and Doxo, at two different concentrations [0,2 μM] and [0,5 μM], alone and in combination. The Muse™ Annexin V & Dead Cell Assay utilizes Annexin V to detect phosphatidylserine (PS) on the external membrane of apoptotic cells. A dead cell marker is also used as an indicator of cell membrane structural integrity, 7-amino-actinomycin D (7-AAD). Briefly, 100 μL of a cell suspension (1 × 10^4^ cells/mL) was mixed with 100 μL of Muse™ Annexin V & Dead Cell Reagent and incubated for 20 min. at room temperature in dark. The results, automatically displayed, were analyzed with “Muse 1.4 Analysis” software for data acquisition.

### Cell cycle analysis

Cell cycle progression was evaluated after 48 h exposure to Den [30 μg/mL] and Doxo, at two different concentrations [0,2 μM] and [0,5 μM], alone and in combination by a fluorimetric assay on the Muse cell Analyser machine with the “Cell Cycle Assay Kit”. The Muse™ Cell Cycle Assay uses a propidium iodide (PI) staining of DNA content to discriminate and measure the percentage of cells in each cell cycle phase (G0/G1, S, and G2/M). The two parameters considered by this assay are cell size and DNA content. Briefly, OS cells were fixed in 70% ice-cold ethanol at 4°C over-night, washed and incubated with Cell Cycle reagent for 30 min. at room temperature in the dark. The results, automatically displayed, were analyzed with “Muse 1.4 Analysis” software for data acquisition.

### Scratch assay

MG-63 and U-2 OS cells were seeded in a six-well plate (500.000 cells per well) and incubated 48 h at 37°C in a humidified atmosphere with 5% CO2 to let them attach in monolayer in the wells. A single scratch was made at the center of a 70–80% confluent well with a 200 μL sterile pipette tip. Each well was washed with PBS and added with fresh media containing Den [30 μg/mL] and Doxo, at two different concentrations [0,2 μM] and [0,5 μM], alone and in combination. Fresh medium without drug was used as control. The migration capacity has been measured in terms of reduction of the scratched area (nm^2^) 48 h after the scratch compared, to the same well at time 0. Hence scratched area in non-treated cells at T0 has been compared vs non-treated cells scratched area at T48 and treated cells at T0 vs treated cells at T48. The wider is the area 48 h later, the lower is the migration observed in the well. Images were taken 48 h later with an AE2000 microscope (Motic) and analyzed with Motic Images plus 2.0 Software, in order to evaluate the cell’s ability to invade the scratched area.

### ELISA assay

The amount of RANK-L released in supernatants from MG63 and U-2 OS cell cultures, was measured using a commercially available Human RANK-L ELISA Kit (LSBio, Inc. Seattle), according to the manufacturer’s instructions. Briefly, a microplate was coated with monoclonal antibody specific to the ligand. Standards and supernatants were pipetted into the wells of the microplate. A positive control was obtained by pipetting only the standard into the wells. A negative control was obtained by pipetting the standard and cell culture supernatants into non-coated wells. After the plate was washed, enzyme-linked polyclonal antibody specific for RANK-L was added to the wells. The reaction was revealed by the addition of the substrate solution. The optical density was measured at a wavelength of 450 nm by using the Tecan Infinite M200 (Tecan, Switzerland) spectrophotometer. RANK-L concentrations (pg/mL) was determined against a standard concentration curve.

### Statistical analysis

Results are expressed as means ± S. D. All the experiments were run in technical triplicate. Statistical analyses on all data were performed using one-way analysis of variance, ANOVA followed by Tukey’s post hoc test (StatGraphics Centurion XV. II Software. Adalta, Arezzo, Italy; Statpoint Technologies Inc., VA). A *p* value less or equal than 0.05 (^*^) was considered statistically significant.
